# Pan-transcriptome analysis of pine wilt disease-resistant and susceptible *Pinus* species and a hybrid

**DOI:** 10.3389/fgene.2026.1743952

**Published:** 2026-03-26

**Authors:** Bae Young Choi, Dayoung Lee, Jaegyeong Jung, Yang-Gil Kim, Youn-Il Park, Kyu-Suk Kang, Donghwan Shim

**Affiliations:** 1 School of Liberal Arts and Sciences, Korea National University of Transportation, Chungju, Republic of Korea; 2 Department of Agriculture, Forestry and Bioresources, Seoul National University, Seoul, Republic of Korea; 3 Department of Biological Sciences, Chungnam National University, Daejeon, Republic of Korea; 4 Center for Genome Engineering, Institute for Basic Science, Daejeon, Republic of Korea

**Keywords:** deep learning, pan-transcriptome, phenome, pine wilt disease, *Pinus* species and hybrid

## Abstract

Pine trees, globally distributed and economically vital evergreen conifers, are threatened by pine wilt disease (PWD) attributed to the pine wood nematode (PWN). Many studies have been conducted on phenome and transcriptome profiling in select *Pinus* species upon PWN infection, but a high-throughput phenotyping of PWD progression and transcriptomic analysis across diverse *Pinus* species remains lacking. Here, we developed a deep learning-based phenotyping program to quantify PWD symptoms and conducted a pan-transcriptome analysis using PWD-susceptible (*Pinus densiflora*, *Pinus koraiensis*, *Pinus thunbergii*) and -resistant (*Pinus parviflora*, *Pinus strobus*, *Pinus rigida* × *Pinus taeda*) *Pinus* species and a hybrid. Our results showed severe wilting of leaves within 14 weeks after PWN infection in susceptible species but not in resistant ones. Pan-transcriptomic analysis revealed the upregulation of genes involved in leaf abscission and abscisic acid responses in PWD-resistant taxa, while PWD-susceptible taxa downregulated genes associated with desiccation response after PWN infection. These findings suggest that activating genes involved in water conservation plays a role in mitigating PWD infection in *Pinus* trees. Notably, all five *Pinus* species and one hybrid exhibited upregulation of the *elongation factor Tu receptor* (*EFR*) gene and *pathogenesis-related (PR)-3* gene upon PWN infection, suggesting a potential role of the EF-Tu receptor in detecting PWN invasion and activating the *PR-3* gene. Our study introduces a novel deep learning-based phenotyping program for precise PWD symptom quantification and enhances understanding of the molecular mechanisms underlying PWD resistance. These insights contribute to high-throughput monitoring of PWD progression in *Pinus* forests for disease prevention and facilitate the development of PWD-resistant pine trees.

## 1 Introduction

Pine trees, widely distributed in the northern hemisphere, play crucial roles in forest ecosystems by providing timber, food, and habitat for diverse wildlife species (Schoettle, 2004; Hao et al., 2015), as well as yielding medicinal compounds with antioxidants and antitumor properties (Kwak et al., 2006). Despite their significance in diverse fields, pine forests are significantly threatened by pine wilt disease (PWD) caused by the pine wood nematode (PWN), *Bursaphelenchus xylophilus*. PWD-susceptible *Pinus* species suffer from rapid leaf wilting post-PWN infection, resulting in severe economic and ecological losses (Zhao et al., 2008). The spread of PWD involves intricate interactions between PWNs, pine trees, and insect vectors, long-horned beetles (*Monochamus*). PWNs infest dying trees during beetle oviposition and multiply within beetle larvae. They are subsequently carried by adult beetles to healthy pine trees during beetle feeding, disrupting the water-conducting system in trees and causing wilting and eventual death in infected trees (Mamiya, 1983; Futai, 2013). Since beetles are widely spread over long distances, PWD is exceptionally destructive and highly transmissible (Kim et al., 2020). Therefore, effective and feasible disease monitoring and detection systems in the early stage of a PWD outbreak are crucial for disease management.

PWD has become a significant concern in various countries, including the United States, Canada, Portugal, Spain, Japan, South Korea, China, Taiwan, and others (Back et al., 2024). In South Korea, PWD has spread from southern to northern regions, threatening PWD-susceptible pine trees like *Pinus thunbergii*, *Pinus densiflora*, and *Pinus koraiensis* (Shin, 2008). PWD is one of the most severe forest diseases in South Korea due to over 40% of the forest area comprising PWD-susceptible *Pinus* species (Kwak et al., 2012). In contrast to these susceptible *Pinus* species, certain *Pinus* species and a hybrid, including *Pinus strobus*, *Pinus rigida*, *Pinus taeda*, and *P. rigida* x *P. taeda*, originating from North America, exhibit resistance to PWD (Mamiya, 1983). Despite numerous studies on PWD susceptibility in various *Pinus* species, most rely on observations from natural disease occurrences or greenhouse experiments (Woo et al., 2008; Franco et al., 2011), limiting the ability to quantify the progression of disease symptoms across diverse *Pinus* species.

In contrast to mobile PWN transmitted by beetle vectors, sessile pine trees in the forest are highly vulnerable to disease spread. To cope with pathogen invasion, pine trees undergo extensive genome-wide transcriptional reprogramming. Transcriptome analyses in diverse *Pinus* species have shown altered gene expression levels upon PWN invasion (Lee et al., 2018; Lee et al., 2019; Zhang Y. et al., 2023; Lee et al., 2024), highlighting the importance of understanding transcriptional changes in mitigating PWD symptoms. However, the comprehensive characterization of PWD-responsive genes across diverse *Pinus* taxa has not been studied intensively.

In this study, we conducted PWN infection experiments using five *Pinus* species and one hybrid in a natural forest setting to monitor disease symptoms. Our novel deep learning-based RGB image processing program enabled precise and effective quantification of leaf wilting phenotypes over 20 weeks post-inoculation with PWN. Furthermore, we performed pan-transcriptome analyses on PWD-susceptible (*Pinus densiflora* (Pd), *P. koraiensis* (Pk), *Pinus thunbergii* (Pt)) and resistant (*Pinus parviflora* (Pp), *P. strobus* (Ps), *P. rigida* × *P. taeda* (Pr)) *Pinus* taxa to elucidate common and distinct changes in gene expression associated with PWD susceptibility. Our comprehensive analysis of the diverse pine trees’ responses against PWN infection contributes to assisting in high-throughput monitoring of PWD progression in *Pinus* forests and facilitating the development of PWD-resistant pine tree varieties.

## 2 Materials and methods

### 2.1 Experimental materials and PWN inoculation

The experimental design and detailed information on five *Pinus* species and one hybrid are described in Kim et al. (2023). Twenty same-aged seedlings from each of the five *Pinus* species and one hybrid were used for PWN inoculation experiment (Supplementary Table S1). Species-specific age differences were necessary to ensure comparable stem thickness at the time of nematode inoculation. Pine seedlings were obtained from multiple sources. Seeds of *P. densiflora* were collected in 2016 from the Anmyeondo Seed Orchard managed by the Korea Seed and Variety Service and sown in 2017 in a greenhouse at the Chilbosan Experimental Forest, Seoul National University, South Korea. The seedlings were subsequently transplanted to a field plot at the same institute in 2019. Seedlings of *P. rigida* × *P. taeda* were produced using seeds collected from mature trees maintained at the Forest Bioresources Department of the National Institute of Forest Science, South Korea and were raised in field plots at the same institute. The remaining four pine species were purchased from commercial nurseries; among them, *P. parviflora* seedlings were grafted plants using *P. thunbergii* as the rootstock. On March 30 and April 2, 2021, the trees were transplanted to the Chilbosan experimental forest, Seoul National University, South Korea. Following a 3-month adaptation period in the field, the height and root collar diameter of the trees were measured (Supplementary Table S1).

**FIGURE 1:**
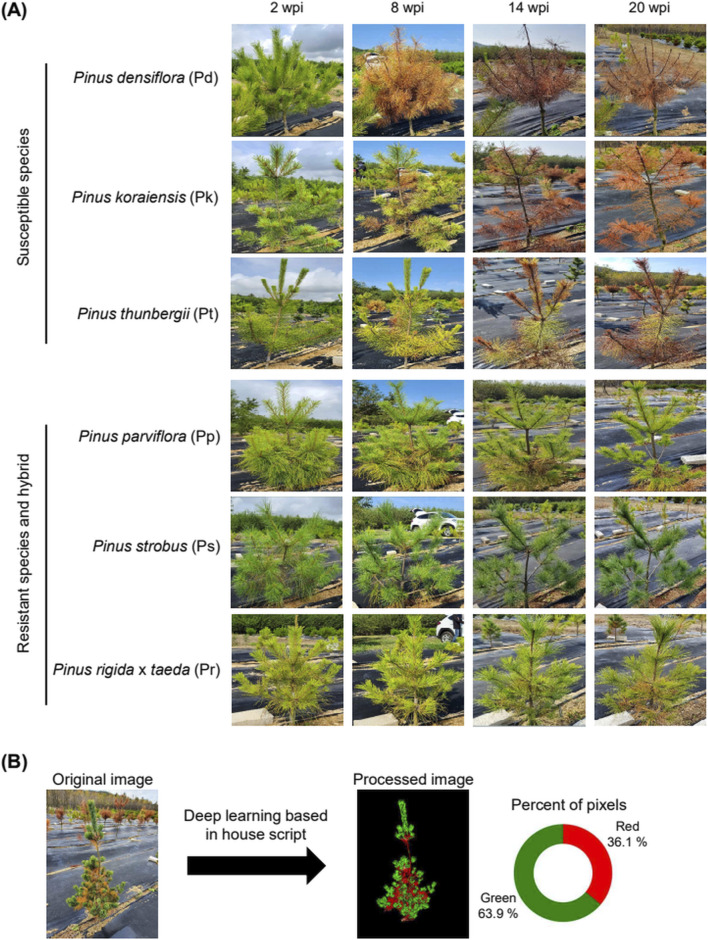
Three *Pinus* species (Pd, Pk, Pt) are susceptible while the other two Pinus species and one hybrid (Pp, Ps, Pr) are resistant to PWN infection. **(A)** Representative RGB photographs of five Pinus species and one hybrid weeks post-inoculation (wpi) of PWN. Five- to eight-year-old pine trees in the test field were used for PWN inoculation. **(B)** Data processing of raw images to distinguish wilted leaves from healthy leaves. Red pixels represent wilted leaves, whereas green pixels represent healthy leaves in trees.

For the PWN inoculation experiment, *Botrytis cinerea*, the feed for PWN, was first cultivated on potato dextrose agar (PDA) medium prepared with Difco™ Potato Dextrose Agar (BD, United States) solution (39 g∙l^−1^) at 25 °C for 7 days. The resulting PDA medium filled with *B. cinerea* was used to grow PWNs, *B. xylophilus*, at 25 °C for 7 days. PWNs isolated from the medium using the Baermann funnel method were used for inoculation (Baermann, 1917; Burkhardt and Day, 2016). PWN injections were conducted on 25 June 2021, during the middle of summer, when PWN-spreading beetles are known to be active in South Korea (Park et al., 2014). The stems of each tree, positioned 10 cm above the ground, were vertically wounded, and then 100 μL of distilled water containing 10,000 PWNs were injected into the cambium layer of the trees. For control conditions, 100 μL of distilled water without PWNs was injected. The health conditions of the leaves were monitored by capturing RGB images at 2, 8, 14, and 20 weeks post-inoculation with PWN (Figure 1A). Experimental and control plants were spatially co-located within the same field plot and managed under identical irrigation regimes. Since pine wilt disease primarily affects the vascular system (Mamiya, 1983; Futai, 2013), and the cambial region represents the primary site of nematode colonization and host defense signaling, for pan-transcriptome analysis, cambium tissues were collected from three biological replicates of each of the five *Pinus* species and one hybrid at 0, 2, and 4 weeks post-inoculation with PWN. The transcriptomic replicates were obtained from the same individual, and the resulting data were processed bioinformatically as a single sample.

### 2.2 RGB leaf segmentation using deep learning programs

To exclusively extract images of pine trees from the original RGB images, image preprocessing was conducted using Stable Diffusion v. 1.3.0 and ControlNet v. 1.1 (Rombach et al., 2022; Zhang L. et al., 2023). Original RGB images were acquired at a resolution of 3,024 × 4,032 pixels and uniformly resized prior to analysis to ensure consistent pixel dimensions across all samples. The preprocessor depth_Midas supported by ControlNet was utilized to acquire a depth map from the original image (Ranftl et al., 2020). The resolution of the acquired image was adjusted to 1/4 of the original, and RGB values were transformed into single values ranging from 0 to 255. Using an in-house Python script (Supplementary Material S1), we excluded the background of images by applying a threshold. The processed image underwent further modification in Photoshop to isolate the desired region of interest. For phenotypic quantification, leaf wilting was assessed using a binary color-based classification approach rather than a continuous color gradient. Healthy and wilted leaf tissues were defined as green and red pixels, respectively, allowing robust and reproducible quantification across taxa with distinct leaf color baselines. For image segmentation of leaves, we adjusted the Hue, Saturation, and Brightness of the processed image, then we calculated the ratio of green to red pixels in the processed images (Figure 1B; Supplementary Figure S1).

### 2.3 Quantification of PWN in infected pine trees

The detailed methodology for detecting PWN in the seedlings is described in Kim et al. (2023). At 24 weeks post-inoculation of PWN, PWNs were isolated from chopped branches and trunks of the PWN-infected trees using the Baermann funnel method (Baermann, 1917; Burkhardt and Day, 2016). A total of five observations per seedling were conducted by applying 10 μL each time, and the isolated PWNs were manually counted using an optical microscope (Olympus SZ61, Tokyo, Japan). After PWN isolation, the remaining branches and trunks were subjected to drying at 80 °C for 7 days to measure their dry mass.

### 2.4 RNA extraction and sequencing

Total RNA from the cambium layer was extracted using the IQeasy™ Plus Plant RNA Extraction Mini Kit (iNtRON, Seongnam-si, Gyeonggi-do, South Korea). RNA quantity and purity were assessed with the Agilent 4200 TapeStation (Agilent Technologies, Santa Clara, CA, United States). For library construction, 500 ng of total RNA per sample with an RNA integrity number (RIN) ≥8.0 was used. Thirty cDNA libraries were sequenced on the Illumina NovaSeq 6000 platform (Illumina, San Diego, CA, United States), generating 100 bp paired-end reads. Library preparation and sequencing were performed at LabGenomics (Seongnam-si, Gyeonggi-do, Republic of Korea).

### 2.5 *De novo* transcriptome assembly and prediction of coding sequence

To conduct *de novo* transcriptome assembly for the five *Pinus* species and one hybrid, the reads were cleaned using PRINSEQ++ v. 1.2.4 to remove duplicated reads (Cantu et al., 2019). The cleaned reads from all samples for each *Pinus* taxon were merged to assemble a single *de novo* transcriptome reference using the Trinity v. 2.15.1 with default parameters (Grabherr et al., 2011). The resulting contigs were clustered using CD-HIT v. 4.8.1 with the following parameters: d 0 -p 1 -T 8 -M 16,000 -n 8. Coding regions within the clustered contigs were identified using TransDecoder v. 5.5.0 (http://transdecoder.github.io). Initially, the TransDecoder.LongOrfs tool was utilized to extract the longest open reading frames (ORFs) from the clustered contigs. Subsequently, the best-evaluated ORFs were predicted as unigenes using TransDecoder.Predict tool with two homology datasets generated by the Protein Basic Local Alignment Search Tool (BLASTP) v. 2.12.0+ search of against the SWISSPROT database and PFAM domain prediction using HMMER v. 3.3.2 (Finn et al., 2011; Punta et al., 2011). The overall quality of *de novo* assembled transcriptomes was evaluated using the benchmarking universal single-copy orthologs (BUSCO) program v. 5.4.4 (Simão et al., 2015).

### 2.6 Transcript quantification and DEG identification

The same RNA sequencing (RNA-Seq) reads used for *de novo* transcriptome assembly were employed for transcript quantification and differential expression analysis. Raw reads were cleaned using PRINSEQ-lite v. 0.20.4 with the following parameters: min_len 50; min_qual_score 10; min_qual_mean 20; derep 14; trim_qual_left 20; trim_qual_right 20 (Schmieder and Edwards, 2011). The cleaned read pairs were aligned to each *de novo* assembled transcriptome using Bowtie2 v. 2.3.5.1 with default parameters (Langmead and Salzberg, 2012). RSEM v. 1.3.0 was used to quantify reads and perform count normalization, resulting in Trimmed Mean of M-values (TMM)-normalized transcripts per million (TPM) values for each transcript (Li and Dewey, 2011). Differential expression analysis was conducted using EdgeR v. 3.16.5 to estimate the negative binomial dispersion across samples (Robinson et al., 2009). Unigenes with a fold change >2 and a *p-value* < 0.05 were determined to be differentially expressed genes (DEGs). Transcriptomic changes were strictly evaluated by contrasting the infected conditions (2 and 4 weeks post-inoculation) against their respective 0-week healthy baseline controls for each taxon, ensuring that the reported differential expression is directly attributable to pathogen exposure.

### 2.7 Functional annotation and network analysis

Functional annotation of unigenes was conducted by searching for sequence similarities between *Pinus* unigenes and the *Arabidopsis thaliana* protein database using BLASTP. Given that *de novo* transcriptome assembly from short-read RNA-Seq data inherently produces partial transcripts, homology was determined based on a stringent e-value threshold of 1 × 10^−5^, maximizing the annotation of conserved functional domains. For Gene Ontology (GO) enrichment analysis, homologous *A. thaliana* genes for all DEGs were analyzed using DAVID (Huang et al., 2009; Sherman et al., 2022). A functional gene network was constructed using GeneMania software (Warde-Farley et al., 2010), utilizing the *A*. *thaliana* database to search for both physical and predicted interactions between *Arabidopsis* homologs of *Pinus* unigenes. The resulting functional gene network was visualized using Cytoscape software v. 3.9.1 (Shannon et al., 2003). Orthologous gene groups across the five *Pinus* species and one hybrid and *A. thaliana* were identified using OrthoFinder v. 2.5.5 (Emms and Kelly, 2019). The gene tree representing the PR-3 orthogroup was visualized using Interactive Tree of Life (iTOL) v.7 (Letunic and Bork, 2024).

## 3 Results

### 3.1 Deep learning-based PWD symptom analysis in five *Pinus* species and one hybrid

**FIGURE 2:**
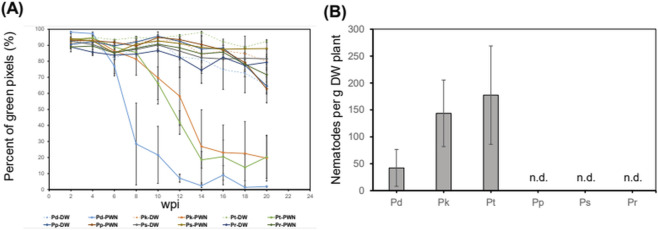
Percentage of green pixels and PWN density of five *Pinus* species and one hybrid. **(A)** Percentage of green pixels in RGB photographs of five *Pinus* species and one hybrid for the indicated weeks post-inoculation of PWN (DW represents water-treated control). Data are shown as the mean ± SEM; N = 3. **(B)** PWN density of five *Pinus* species and one hybrid for 24 weeks post-inoculation of PWN. Data are shown as the mean ± SEM; N = 3.

To assess the progression of PWD in five *Pinus* species and one hybrid, we conducted RGB imaging of trees after inoculation of 10,000 PWN into the cambium layer of trees (Figure 1A). Over a 20-week period post-inoculation, leaves in susceptible species exhibited rapid wilting, contrasting with minimal wilting observed in resistant species and a hybrid. However, relying solely on visual inspection using RGB picture data proved inadequate for precise quantification of wilting symptoms across the diverse *Pinus* species. We therefore developed a deep learning-based image processing program designed to isolate the target pine tree by eliminating background noise and calculating the ratio of green to red pixels, representing healthy and wilted leaves, respectively (Figure 1B). Using this program, we observed a more rapid decline in the percentage of green pixels in PWN-injected Pd compared to PWN-injected Pt and Pk, as well as water-injected control treatments (Figure 2A). This finding indicates Pd as the most susceptible species to PWD among the five *Pinus* species and one hybrid. Conversely, in resistant species and a hybrid, the percentage of green pixels in PWN-injected trees was comparable to those in water-injected controls, indicating that the health status of leaves in two PWD-resistant *Pinus* species and one hybrid was not altered by PWN injection (Figure 2A).

Furthermore, PWN density measurement at 24 weeks post-inoculation revealed a lower PWN density in Pd compared to Pk and Pt, with no detectable PWN in the two PWD-resistant *Pinus* species and one hybrid (Kim et al., 2023) (Figure 2B). These results indicate that, compared with resistant *Pinus* species and a hybrid, PWN proliferated more actively within susceptible species. In contrast, in the resistant species and a hybrid, both horizontal and vertical migration of PWN were restricted, thereby limiting the spread of PWN from the cambium to the xylem (Li et al., 2023). However, the average density of PWN within the xylem may vary depending on the sampling location and timing, which could influence the interpretation of species-specific differences in symptom severity. Together, our deep learning-based analysis of leaf wilting symptoms facilitated precise quantification, thereby ranking the susceptibility of *Pinus* species and a hybrid to PWD: Pd showed the highest susceptibility, Pt and Pk displayed intermediate susceptibility, whereas the other two species and one hybrid were classified as resistant, providing a phenotype-based framework for interpreting transcriptomic variation.

### 3.2 *De novo* transcriptome assembly of five *Pinus* species and one hybrid and identification of unigenes

To decipher the genome-wide transcriptional reprogramming in response to PWN infection among PWD-resistant and susceptible *Pinus* species and a hybrid, we conducted RNA-Seq analyses using cambium layers from three independent trees of each taxon sampled before and after inoculation with PWNs at 0, 2, and 4 weeks (Supplementary Table S2). Following the removal of low-quality, short, and duplicated sequences, we obtained cleaned read pairs from each of the five *Pinus* species and one hybrid (Supplementary Table S3). These cleaned read pairs were used to assemble *de novo* transcriptomes of these taxa using the Trinity program (Grabherr et al., 2011). From the assembled contigs, we selected 44,492, 48,860, 48,850, 55,604, 39,547, and 44,914 unigenes using TransDecoder program, with an average GC content of 43.3% and an average N50 value of 2,160 bp (Table 1). To assess the quality of the transcriptome assembly, we employed BUSCO v5 (Benchmarking Universal Single-Copy Orthologs) (Simão et al., 2015). The BUSCO analysis revealed that the percentage of complete sequences in the embryophyta_odb10 database exceeded 80.1% for all six taxa (Table 1; Supplementary Table S5), indicating the high quality of the unigene curation for studying the changes at the transcript level in response to PWD.

**TABLE 1:** Statistics of *De novo* transcriptome assembly of five *Pinus* species and one hybrid.

Species/Hybrid		Pine wilt disease	Transcriptome assembly			
			Number of transcripts	Percent GC, %	N50, bp	Complete BUSCOs (transcriptome), %
*Pinus densiflora*	Pd	Susceptible	44,492	45.86	2,047	80.1
*Pinus koraiensis*	Pk	Susceptible	48,860	42.03	2,459	88.5
*Pinus thunbergii*	Pt	Susceptible	48,850	43.16	2,103	88.4
*Pinus parviflora*	Pp	Resistant	55,604	44.13	1,920	87.8
*Pinus strobus*	Ps	Resistant	39,547	42.63	2,173	88.7
*Pinus rigida* × *Pinus taeda*	Pr	Resistant	44,914	42.09	2,258	87

### 3.3 Pan-transcriptome analysis reveals common responsive DEGs in PWD-resistant and susceptible *Pinus* species and a hybrid

To dissect the molecular mechanisms underlying PWD in *Pinus* species and a hybrid, we conducted a comprehensive analysis of genome-wide transcriptional changes in PWD-resistant and–susceptible *Pinus* species and a hybrid. Clean read pairs were aligned to the *de novo* assembled transcriptome, achieving an overall mapping rate exceeding 72% across the five *Pinus* species and one hybrid (Supplementary Table S2). For comparative analysis of the RNA-Seq data across the five *Pinus* species and one hybrid, DEGs (fold change >2, *p-value* < 0.05) identified from *de novo* assemblies were mapped to *A. thaliana* homologs. Each *Pinus* unigene was then annotated using the nomenclature of its homologous *Arabidopsis* gene.

**FIGURE 3:**
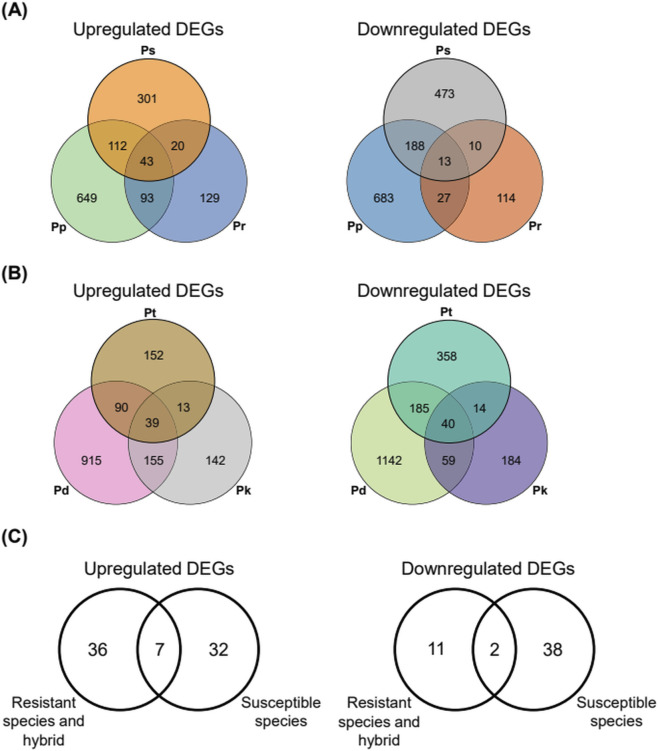
Overview of the DEGs in response to PWN inoculation in two PWD-resistant *Pinus* species and one hybrid (Ps, Pp, Pr) and three *Pinus* PWD-susceptible species (Pd, Pk, Pt). The *Arabidopsis* genes homologous to the *Pinus* unigenes were used to compare DEGs. The Venn diagram represents upregulated and downregulated DEGs in pine trees at 2 and 4 weeks post-inoculation of PWN compared to 0 week. **(A)** PWD-resistant species and hybrid (Ps, Pp, Pr); **(B)** PWD-susceptible species (Pd, Pk, Pt). **(C)** The Venn diagram represents commonly upregulated and downregulated DEGs in five *Pinus* species and a hybrid at 2 and 4 weeks post-inoculation of PWN compared to 0 week.

In PWD-resistant *Pinus* species and a hybrid (Ps, Pp, Pr), we observed the upregulation of 476, 897, and 285 DEGs, with 684, 911, and 164 DEGs downregulated in Ps, Pp, and Pr, respectively, at 2 weeks and 4 weeks post-inoculation of PWN compared to 0 weeks. Among these DEGs, 43 and 13 DEGs were commonly upregulated and downregulated, respectively, in two PWD-resistant *Pinus* species and one hybrid (Figure 3A). In PWD-susceptible *Pinus* species (Pt, Pd, Pk), we identified the upregulation of 294, 1,199, and 349 DEGs, while 597, 1,426, and 297 DEGs were downregulated in Pt, Pd, and Pk, respectively, at 2 weeks and 4 weeks post-inoculation of PWN compared to 0 weeks. Among these DEGs, 39 and 40 DEGs were commonly upregulated and downregulated, respectively, in two PWD-resistant *Pinus* species and one hybrid (Figure 3B).

Considering the distinct leaf wilting phenotypes between PWD-susceptible and resistant *Pinus* species and a hybrid, we hypothesized that DEGs specific to PWD-susceptible *Pinus* species might contribute to leaf wilting, while those specific to PWD-resistant *Pinus* species and a hybrid might confer resistance to leaf wilting. Among the DEGs commonly upregulated in PWD-resistant and susceptible *Pinus* species and a hybrid, 36 and 32 DEGs were specifically upregulated in PWD-resistant and susceptible trees, respectively. In commonly downregulated DEGs, 11 and 38 DEGs were specifically downregulated in PWD-resistant and susceptible *Pinus* species and a hybrid, respectively. Notably, 7 and 2 DEGs were commonly upregulated and downregulated, respectively, in both PWD-resistant and susceptible *Pinus* species and a hybrid (Figure 3C; Supplementary Figures S2 and S1).

Taken together, consistent with the rapid and severe wilting phenotypes observed in susceptible species, a larger number of DEGs and pronounced species-specific transcriptional responses were detected following PWN inoculation. In particular, the highest numbers of both upregulated and downregulated DEGs were identified in Pd, the most susceptible species. In contrast, although resistant species and a hybrid exhibited minimal changes in leaf greenness, a substantial number of DEGs were still regulated, indicating the activation of effective transcriptional responses to mount defense against PWN infection. Moreover, both resistant and susceptible species and a hybrid shared commonly upregulated and downregulated DEGs, suggesting that common pathways underlie responses to PWN infection across different *Pinus* species and a hybrid, irrespective of their susceptibility. These findings suggest the existence of conserved PWD responses in the *Pinus* genus regardless of species-level differences.

### 3.4 Genes involved in water conservation are likely to contribute to leaf wilting symptoms upon PWD infection

**FIGURE 4:**
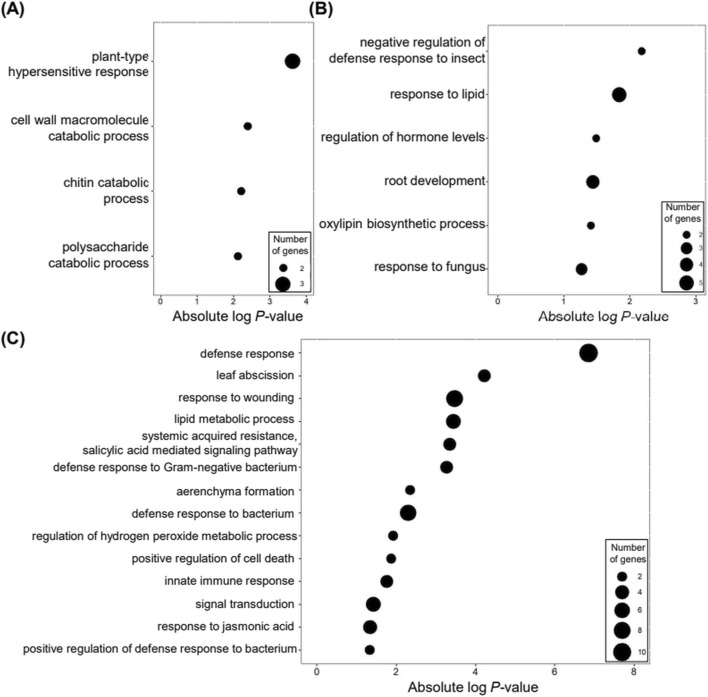
GO analysis of upregulated DEGs at 2 and 4 weeks post-inoculation of PWN compared to 0 week. The *Arabidopsis* genes homologous to the *Pinus* genes were used for GO analysis. The bubble plot representation of GO terms in the biological process category has a *P*-value < 0.05. The size of the bubbles represents the number of genes in GO terms. The X-axis represents the absolute log-transformed *P*-value of the GO terms. **(A)** 7 commonly upregulated DEGs in five *Pinus* species and one hybrid; **(B)** 32 specifically upregulated DEGs in three PWD-susceptible *Pinus* species (Pd, Pk, Pt); **(C)** 36 specifically upregulated DEGs in two PWD-resistant *Pinus* species and one hybrid (Ps, Pp, Pr).

To identify the biological processes enriched in the upregulated DEGs in response to PWD progression, we performed GO analysis using *Arabidopsis* homologs of the *Pinus* unigenes. In the 7 DEGs upregulated in all five *Pinus* species and one hybrid (Figure 3C), we found that DEGs were highly associated with plant-type hypersensitive-response, cell wall macromolecule catabolic process, chitin catabolic process, and polysaccharide catabolic process (Figure 4A). Among the 7 DEGs (Supplementary Table S4), *EFR* and *PR-3* have been studied for their roles in defense responses against pathogens (Zipfel et al., 2006; Qiu et al., 2023). These findings indicate a common induction of genes involved in defense responses to pathogens across *Pinus* species and a hybrid, irrespective of their susceptibility to PWD, at the early stage of PWN infection.

To assess whether such responses reflect broader conservation in gene content, we examined orthogroup sharing across the five *Pinus* species and one hybrid and *A. thaliana* (Supplementary Figure S4). The largest intersection comprised 8,269 orthogroups shared by all species, and the next largest comprised 4,769 orthogroups shared by five *Pinus* species and one hybrid, indicating substantial conservation of gene families across the sampled pines. Consistent with this, a phylogenetic analysis using the PR-3 orthologs showed that differentially expressed unigenes from both resistant (Pp, Ps) and susceptible (Pd, Pk) species clustered within the same PR-3 orthogroup (Supplementary Figure S5), supporting conservation of this defense-related gene family across phenotypic classes. In contrast, orthogroups uniquely present in only the resistant set or only the susceptible set were rare (below the plotting threshold of 200 orthogroups in Supplementary Figure S4), suggesting that the resistant–susceptible differences are unlikely to be explained by large-scale gene presence/absence differences, but may instead reflect regulatory and/or expression-level variation.

Further analysis of the 32 DEGs specifically upregulated in PWD-susceptible *Pinus* species revealed GO enrichment in the negative regulation of defense response to insects, response to lipid, regulation of hormone levels, root development, oxylipin biosynthetic process, and response to fungus terms (Figure 4B). In contrast, we identified a total of 14 significant GO terms for DEGs specifically upregulated in PWD-resistant *Pinus* species and a hybrid (Figure 4C). These included genes involved in defense hormones such as salicylic acid and jasmonic acid, immune responses, lipid metabolism, and defense responses, suggesting their role in overcoming PWN invasion in PWD-resistant *Pinus* species and a hybrid. Notably, genes associated with leaf abscission and aerenchyma formation were specifically upregulated in PWD-resistant *Pinus* species and a hybrid, potentially contributing to leaf protection against wilting during PWD progression.

**FIGURE 5:**
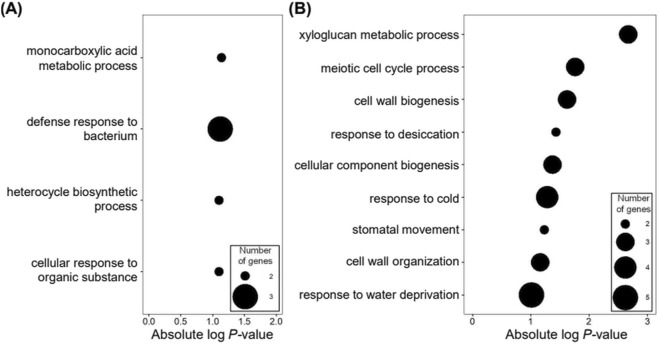
GO analysis of downregulated DEGs at 2 and 4 weeks post-inoculation of PWN compared to 0 week. The *Arabidopsis* genes homologous to the *Pinus* genes were used for GO analysis. GO terms in the biological process category were visualized on the bubble plot. The size of the bubbles represents the number of genes in GO terms. The X-axis represents the absolute log-transformed *P*-value of the GO terms. **(A)** 11 specifically downregulated DEGs in two PWD-resistant *Pinus* species and one hybrid (Ps, Pp, Pr); **(B)** 38 specifically downregulated DEGs in three PWD-susceptible *Pinus* species (Pd, Pk, Pt).

For downregulated DEGs in response to PWD, GO term identification was limited due to the small number of commonly downregulated DEGs in all five *Pinus* species and one hybrid (Figure 3C). However, GO analysis revealed enrichment of DEGs specifically downregulated in PWD-resistant *Pinus* species and a hybrid in monocarboxylic acid metabolic process, defense response to bacterium, heterocycle biosynthetic process, and cellular response to organic substance (Figure 5A). Conversely, DEGs specifically downregulated in PWD-susceptible *Pinus* species were enriched in metabolic processes related to cell wall biogenesis and response to cold (Figure 5B). Notably, DEGs involved in response to desiccation, stomatal movement, and response to water deprivation were specifically downregulated in PWD-susceptible *Pinus* species, suggesting their potential role in the severe leaf wilting phenotype observed in response to PWD. Furthermore, the most highly enriched GO term using upregulated DEGs was negative regulation of defense response to insect (Figure 4B), suggesting that defense responses to PWN might not be properly induced in PWD-susceptible *Pinus* species. Detailed analysis of this category revealed that *JOX2* homologs were significantly upregulated in susceptible species but not in resistant ones (Supplementary Figure S6). These species-specific induction patterns suggest JOX2 as a promising genetic marker for assessing PWD symptoms. Taken together, our pan-transcriptome analysis revealed a significant upregulation of genes involved in water conservation in PWD-resistant *Pinus* species and a hybrid but downregulation in PWD-susceptible *Pinus* species at the early stage of PWN infection. These findings suggest that the induction of genes related to water conservation may be crucial for protecting leaves during the early period of PWD, thereby conferring subsequent resistance to PWN invasion.

## 4 Discussion

Early diagnosis of PWD is crucial for effective management of healthy pine forests, as it allows for timely intervention to prevent disease spread. Recently, PWD diagnosis studies have highly relied on analyzing images obtained from unmanned aerial vehicles (UAVs) based on natural disease occurrences (Wu et al., 2021; Yu et al., 2021). However, these approaches often lack the resolution for precise individual tree assessment and struggle with environmental noise. To address these limitations, we developed a semi-automated, deep learning-based phenotyping pipeline capable of analyzing pine trees within complex field environments. Our approach utilizes Midas depth estimation to automatically separate the target tree from complex backgrounds. This method effectively filters out overlapping canopies, human operators, variable ground surfaces, and natural shadows that typically hinder field phenotyping. By eliminating the need for laborious pixel-level manual masking, our pipeline allows for scalable, high-throughput analysis using simple RGB images. This approach is cost-effective and accessible compared to LiDAR or hyperspectral imaging, which require specialized equipment and operational expertise.

Our dataset offers distinct advantages for quantifying the PWD progression. Unlike UAV-based image data collected from natural forests, which may include images of naturally wilted trees caused by environmental or other biological stresses (You et al., 2022), our dataset is derived exclusively from experimentally infected trees in a field environment. This experimental design minimizes false positives, ensuring that the monitored symptoms are specifically attributable to PWD across diverse *Pinus* species and a hybrid. We biologically validated this specificity by successfully isolating PWNs from the three susceptible species at 24 weeks post-inoculation (Figure 2B). Furthermore, the high temporal resolution of our dataset captures the dynamic trajectory of PWD symptom development. By monitoring trees for 20 weeks post-inoculation of PWN in pine trees (Figures 1A, 2A), we documented the precise chronological progression of PWD in three PWD-susceptible *Pinus* species (Pd, Pk, Pt). This time-series dataset is valuable for tracking disease development and provides foundational insights for developing robust species-specific PWD diagnostic tools.

A systems genomics–based integrative multi-omics approach enables a more comprehensive understanding of complex genotype–phenotype relationships (Nachtomy et al., 2007; Ritchie et al., 2015). In addition, multispecies transcriptomic data allow comparative exploration of gene loci exhibiting expression-level changes across the entire transcriptome (Harrison et al., 2012). The integration of image-based phenotypic analysis and transcriptomic analysis in this study provides important insights into species-specific defense strategies against PWD. Deep learning–based quantitative analysis of leaf wilting revealed distinct differences in symptom severity among *Pinus* species and a hybrid, enabling objective classification into susceptible, moderately susceptible, and resistant groups. The greater phenotypic variance observed among highly susceptible *Pinus* species is likely to reflect inherent biological heterogeneity in disease progression rather than experimental noise. In susceptible hosts, variability in pathogen establishment processes, such as the latent and infectious periods, as well as differences in within-host spread following establishment, can lead to increased phenotypic heterogeneity during disease progression (Burdon et al., 1989). In contrast, resistant hosts effectively restrict early pathogen spread through robust resistance mechanisms against the nematode (Hirao et al., 2012; Modesto et al., 2021), resulting in uniformly mild or absent symptoms across individuals. In addition, these phenotypic differences were accompanied by distinct transcriptomic response patterns, suggesting that visible wilting severity reflects underlying differences in physiological and molecular processes.

At the early stage of PWD, nematodes migrate along the cortex and subsequently invade the xylem of the stem, a process that induces degeneration and necrosis of parenchyma cells (Fukuda, 1997). In other words, the infection of pine trees by PWN triggers the formation of cavitation in the xylem, disrupting water flux and ultimately leading to leaf wilting (Kuroda, 1991). From a physiological perspective, leaf wilting can be regarded as a downstream outcome resulting from the cumulative effects of reduced photosynthesis, disruption of the cambium, production of phytotoxic compounds, and increased respiration rates (Bolla et al., 1984; Fukuda et al., 1992). Genes associated with stress responses, defense signaling, and metabolic regulation identified in this study are likely to influence the severity and timing of visible symptom development by indirectly participating in these processes. The coordinated expression patterns of these genes suggest that phenotypic outcomes are not driven by the effect of a single gene, but rather arise from interactions among multiple defense-related pathways.

In this study, genes associated with hormone regulation, including those related to jasmonic acid (JA), were specifically upregulated in both susceptible and resistant *Pinus* species and a hybrid. Previous studies have reported differential expression of genes encoding JA biosynthetic enzymes in *Pinus pinaster* following PWN inoculation in both susceptible and resistant individuals (Gaspar et al., 2016; Modesto et al., 2021), and upregulation of JA biosynthesis–related genes in susceptible individuals of *Pinus massoniana* (Xu et al., 2012; Xie et al., 2020). These findings suggest that JA plays an essential role in the response to PWN in *Pinus* species and a hybrid and is closely associated with resistance (Modesto et al., 2022). In contrast, metabolic pathways related to cell wall biosynthesis were specifically downregulated in susceptible species. Lignin, a major structural component of the plant cell wall, has been reported to be associated with resistance to pine wilt disease in *Pinus* species. Higher expression levels of genes encoding enzymes involved in lignin biosynthesis were observed in resistant individuals of *P. massoniana*, *P. pinaster*, and *P. thunbergii* (Hirao et al., 2012; Liu et al., 2016; Modesto et al., 2021), suggesting that increased lignin deposition in the cell wall is correlated with resistance. Thus, cell wall reinforcement is associated with enhanced resistance to pathogens (Modesto et al., 2022), and the reduced expression of cell wall biosynthesis–related pathways observed in susceptible species in this study is consistent with these findings.

Genes involved in water conservation were specifically upregulated in PWD-resistant *Pinus* species and a hybrid, but not in PWD-susceptible *Pinus* species upon PWN infection (Figures 4, 5). These results suggest the importance of activating genes involved in water conservation for mitigating PWD symptoms. Previous reports support the importance of water conservation in PWD tolerance in pine trees. The progression of PWD decreases water potential and hydraulic conductivity, likely inducing water-deficient stress in PWD-infected pine trees (Yazaki et al., 2017). In Pt, *P. pinaster*, and *Pinus radiata*, drought stress synergistically decreases the water status in PWN-infected pine trees, facilitating progression of PWD symptoms and increasing mortality rates in these species (Ikeda, 1996; Estorninho et al., 2022). Previous studies have demonstrated that *ABCG40* mediates cellular ABA uptake, which is essential for rapid stomatal closure in response to drought and pathogen attacks (Kang et al., 2010). The specific upregulation of these genes in PWD-resistant species and a hybrid suggests a rapid physiological reprogramming to prevent moisture loss and vascular cavitation immediately following PWN infection. Unlike susceptible species that fail to activate this desiccation-response network, resistant trees appear to effectively utilize ABA signaling pathways to maintain hydraulic conductivity, thereby delaying or preventing the visible wilting phenotype.

Our pan-transcriptome analyses across five *Pinus* species and one hybrid revealed a common response to PWD characterized by the induction of *EFR* and *PR-3* (Figure 3C), suggesting their role in defense responses against PWN invasion. Plants perceive the presence of microbes through the recognition of pathogen-associated molecular patterns (PAMPs) by pattern-recognition receptors (PRRs) localized at the cell surface (Jones and Dangl, 2006; Zipfel, 2008). *Arabidopsis thaliana* can detect a broad range of PAMPs, including fungal chitin as well as bacterial flagellin and elongation factor Tu (EF-Tu) (Gomez-Gomez and Boller, 2000; Zipfel et al., 2006). Perception of flagellin and EF-Tu initiates a suite of downstream immune responses, including the activation of defense-related gene expression and the biosynthesis of defense-associated hormones (Schwessinger and Zipfel, 2008). In this context, EFR functions as the PRR responsible for the recognition of EF-Tu (Nekrasov et al., 2009). The invasion of PWN is accompanied by physical damage to host pine trees, suggesting that PWN-carried bacteria may contribute to PWD development and impact host defense responses (Proença et al., 2017). Inoculation of aseptic PWN does not cause PWD symptoms in pine trees (Han et al., 2003; Zhao et al., 2009), underscoring the importance of defense responses against PWN-carried bacteria in coping with PWD. Therefore, our results suggest that the induction of *EFR*, a representative pattern recognition receptor that recognizes *EF-Tu* from PWN-associated bacteria or nematode-associated PAMPs (Holbein et al., 2016), may play a key role in activating defense responses against external microbes and thereby mitigating the progression of pine wilt disease.

Similarly, the induction of *PR (Pathogenesis-Related)-3*, encoding a chitinase, might be essential for defense against PWN invasion. The PR-3 group comprises a diverse set of chitinase–lysozymes that belong to three distinct classes. These proteins are localized either in the vacuole or in the extracellular space and function in the hydrolysis of structural components of invading microorganisms (Swegle et al., 1992; Stintzi et al., 1993). Since chitin is a main component of nematode eggshell (Fukushige and Futai, 1985), activation of PR-3 may compromise the proliferation of PWN. Consistent with our pan-transcriptome analyses, the induction of *PR-3* transcript levels was required for *A. thaliana* plants to cope with the root-knot nematode *Meloidogyne incognita* (Hamamouch et al., 2011). In Pt and *P. pinaster*, the expression levels of *PR-3* in PWD-resistant cultivars was higher than that in PWD-susceptible cultivars (Nose and Shiraishi, 2011; Modesto et al., 2021). PtPR-3 has been identified as a major target for the PWN virulence factor BxLip-3, which suppresses immune responses in Pt (Qiu et al., 2023). In contrast, it has been reported that although the expression levels of *PR* genes were strongly upregulated during the early stage of PWN infection in Pt seedlings, all seedlings eventually exhibited external disease symptoms (Yamaguchi et al., 2019). This observation suggests that, despite the induction of defense responses upon PWN invasion, these responses may be insufficient to effectively control PWN, thereby resulting in susceptibility. While the functional roles of PR genes have been predominantly characterized in angiosperm models like *Arabidopsis*, their extensive upregulation across diverse *Pinus* species and a hybrid underscores an evolutionarily conserved basal defense mechanism in gymnosperms against nematode invasion. However, as observed in our transcriptomic profiling, the induction of *PR-3* alone is insufficient to halt PWD progression in susceptible species. This implies that while the initial recognition and basal immune response (*via EFR* and *PR-3*) are conserved across the *Pinus* genus, the critical determinant of survival lies in the subsequent capacity to mount parallel physiological defenses, such as the aforementioned robust water conservation and compartmentalization strategies.

Taken together, our results support a model in which the severity of leaf wilting reflects the intensity and coordination of underlying physiological and transcriptomic responses. By integrating quantitative image-based phenotypic analysis with comparative transcriptomic analysis across multiple *Pinus* species and a hybrid, this study presents a pan-transcriptomic analytical framework that simultaneously captures molecular responses and phenotypic outcomes associated with PWD susceptibility and resistance.

## 5 Conclusion

In this study, we developed a novel deep learning-based phenotyping program to decipher the dynamics of PWD-mediated leaf wilting symptoms, which enables precise and effective identification of PWD progression stages. Our pan-transcriptome analysis revealed shared PWD responses among five *Pinus* species and one hybrid, implying the presence of conserved PWD responses in the *Pinus* species and a hybrid. We identified that the activation of genes involved in water conservation might be crucial for alleviating leaf wilting symptoms associated with PWD. Our comprehensive analysis of diverse *Pinus* species and a hybrid upon PWN infection provides valuable insights for understanding the molecular mechanisms underlying PWD resistance and effective management of PWD in pine tree forests. Furthermore, our findings offer a foundation for developing strategies to identify biomarkers for PWD diagnosis and enhance PWD tolerance in pine trees, contributing to the preservation and sustainability of pine tree ecosystems.

## Data Availability

The datasets presented in this study can be found in online repositories. The names of the repository/repositories and accession number(s) can be found below: https://www.ncbi.nlm.nih.gov/, PRJNA1127083.

## Supplementary material

SUPPLEMENTARY FIGURE S1 Schematic of a deep learning based in-house script for distinguishing wilted leaves from healthy leaves using RGB images.

SUPPLEMENTARY FIGURE S2 The expression levels of upregulated unigenes in five *Pinus* species and one hybrid homologous to seven Arabidopsis genes in Figure 3C.

SUPPLEMENTARY FIGURE S3 The expression levels of downregulated unigenes in five *Pinus* species and one hybrid homologous to two Arabidopsis genes in Figure 3C.

SUPPLEMENTARY FIGURE S4 UpSet plot showing intersections of orthogroups among five *Pinus* species and one hybrid and *Arabidopsis thaliana*.

SUPPLEMENTARY FIGURE S5 Phylogenetic analysis of AtPR-3 orthologs across five *Pinus* species and one hybrid.

SUPPLEMENTARY FIGURE S6 The expression levels of unigenes in five *Pinus* species and one hybrid homologous to *Arabidopsis thaliana* JOX2 (AtJOX2).

SUPPLEMENTARY TABLE S1 Summary of five *Pinus* species and one hybrid individuals used in this study.

SUPPLEMENTARY TABLE S2 Summary of short-read sequencing results.

SUPPLEMENTARY TABLE S3 Summary of cleaned read pairs.

SUPPLEMENTARY TABLE S4 List of *Arabidopsis* homologs of *Pinus* DEGs that commonly upregulated in all five *Pinus* species and one hybrid upon PWN infection.

SUPPLEMENTARY TABLE S5 Statistics for transcriptome assessment using BUSCO (embryophyta).

SUPPLEMENTARY DATA S1 Python script for image background exclusion using thresholding.
