# Supplementary figures and images for: Pan-transcriptome analysis of pine wilt disease-resistant and susceptible Pinus species and a hybrid
Source: Front Genet. 2026 Mar 26;17:1743952. doi: 10.3389/fgene.2026.1743952 (PMC13061383; doi:10.3389/fgene.2026.1743952)

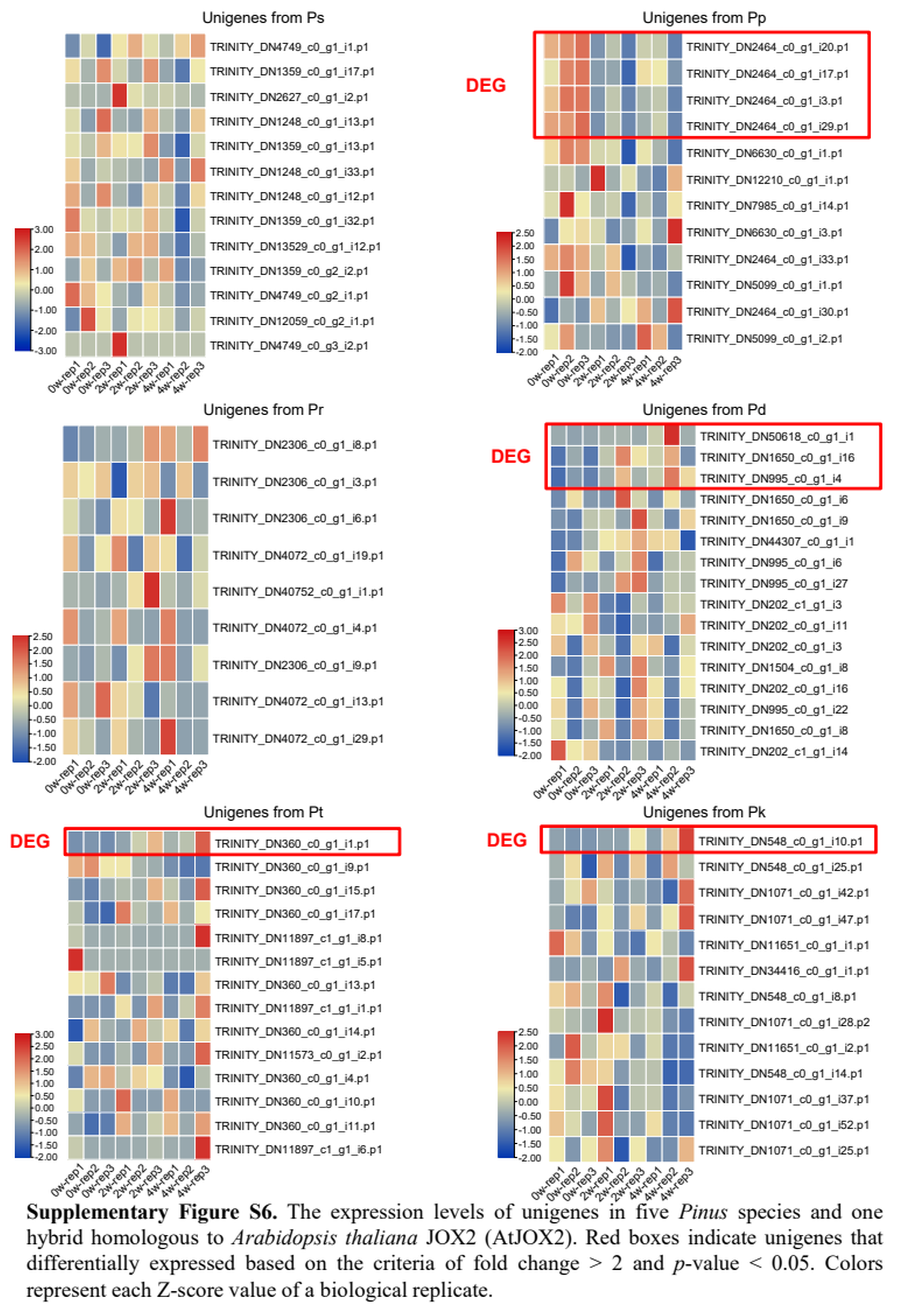

Supplement: Supplementary file 1 [file Image6.tif]

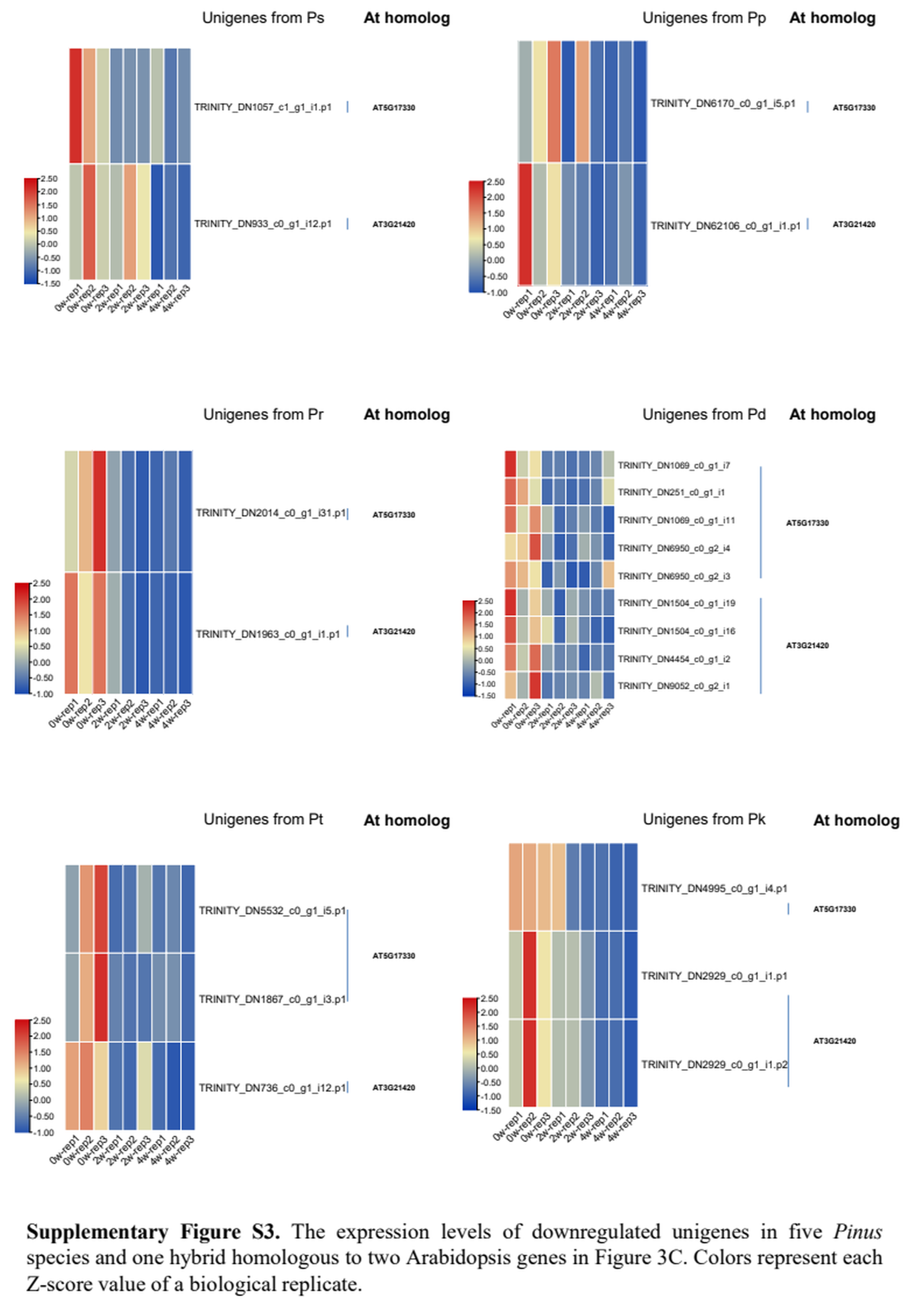

Supplement: Supplementary file 2 [file Image3.tif]

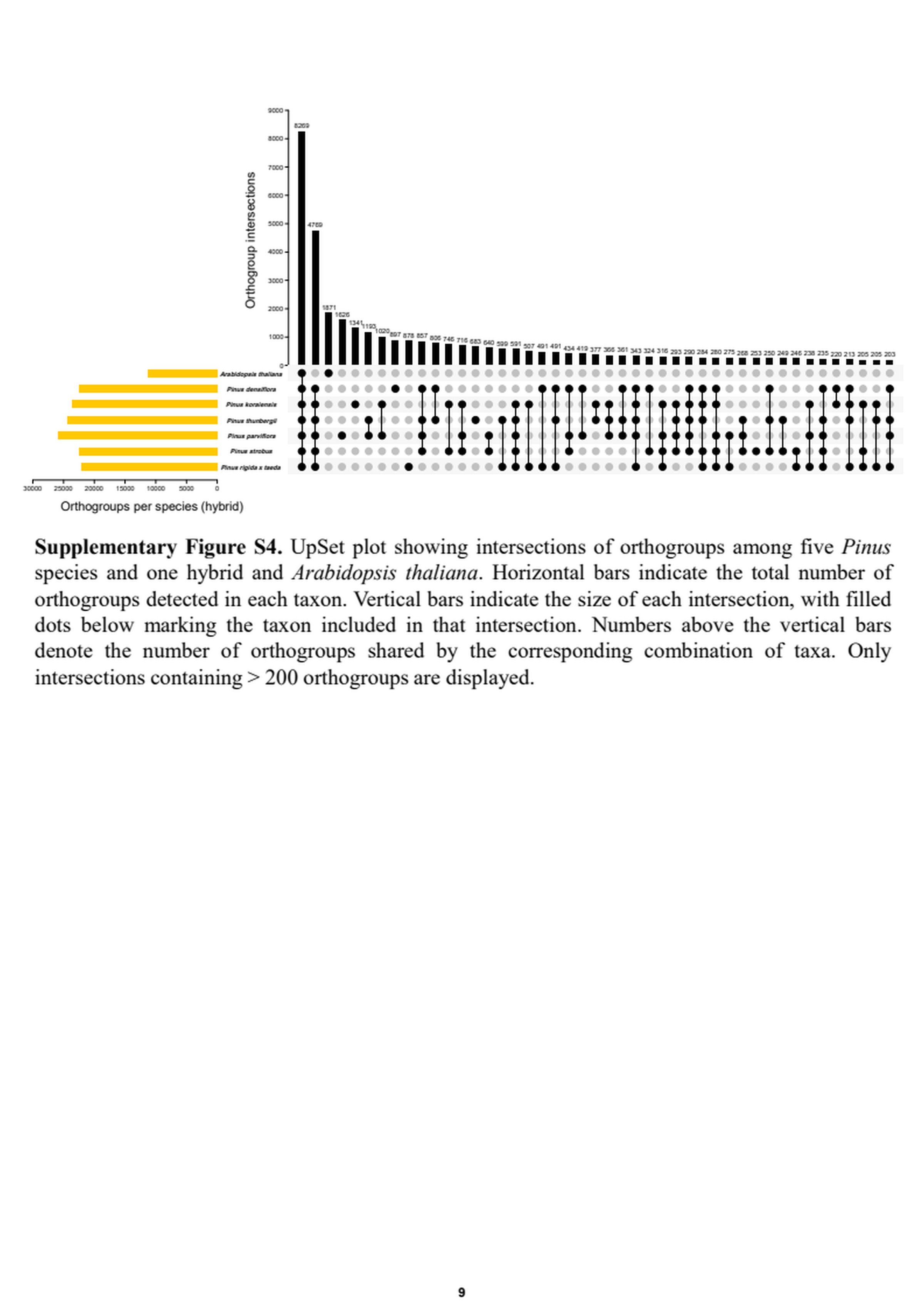

Supplement: Supplementary file 3 [file Image4.tif]

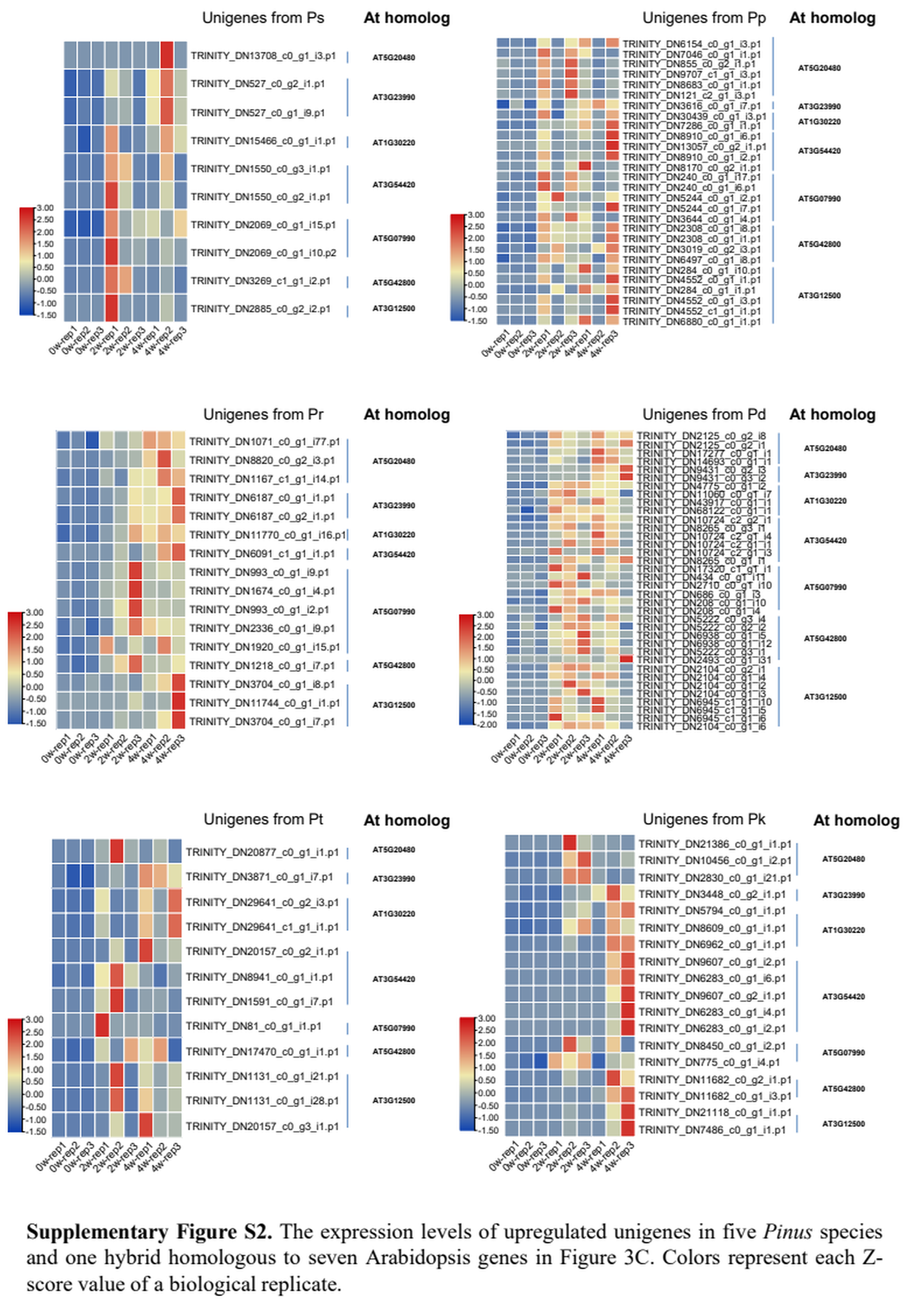

Supplement: Supplementary file 5 [file Image2.tif]

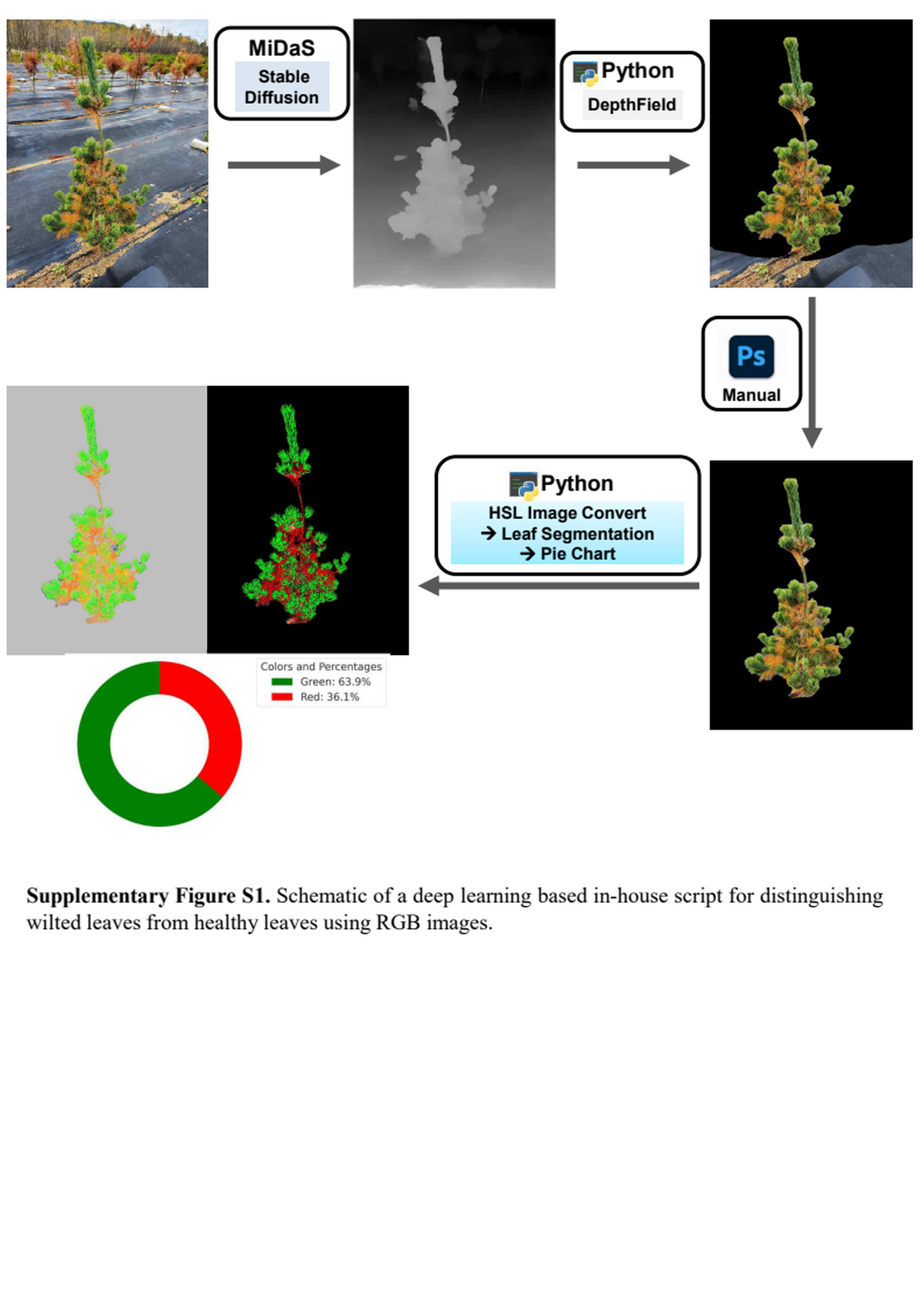

Supplement: Supplementary file 6 [file Image1.tif]

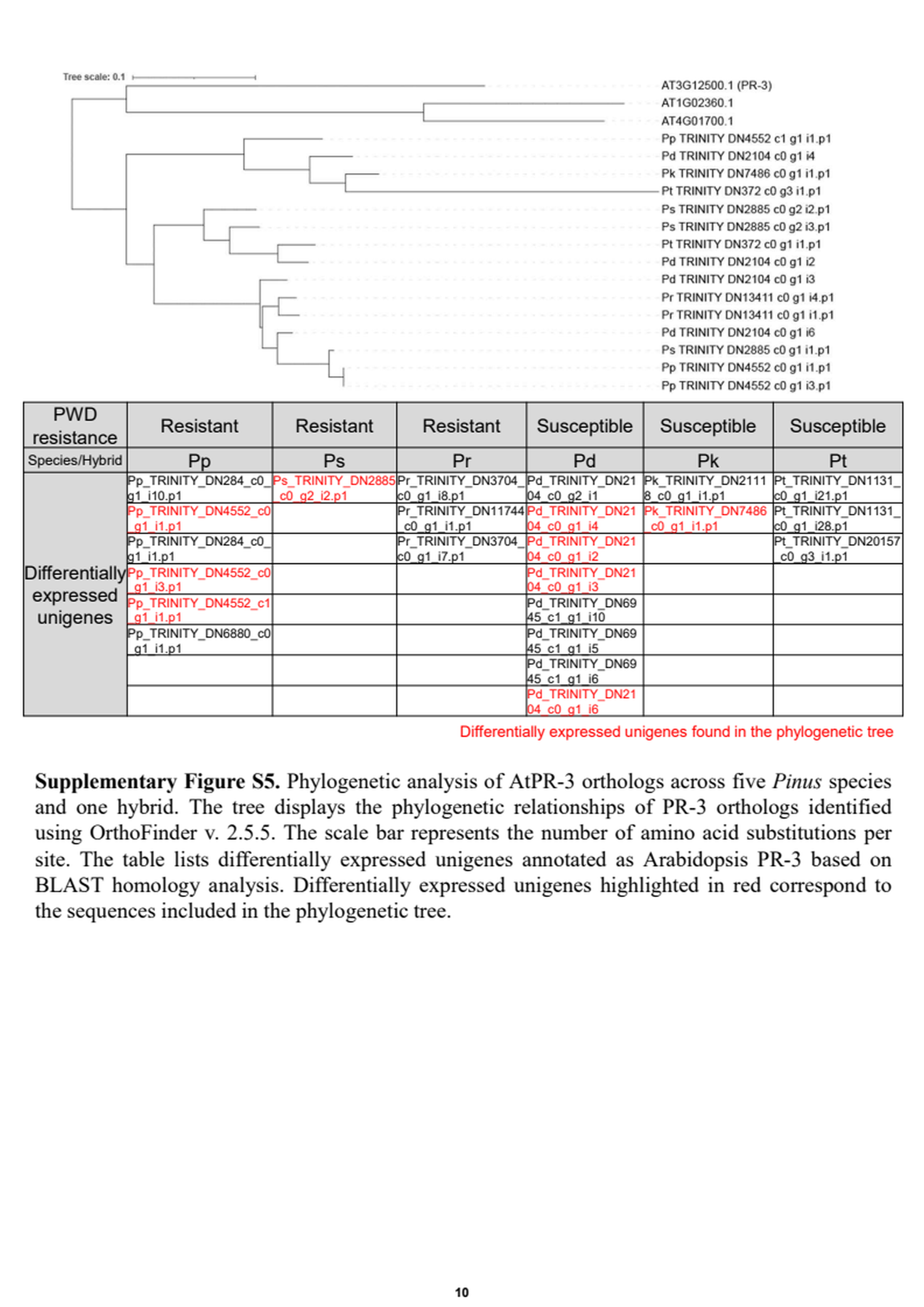

Supplement: Supplementary file 8 [file Image5.tif]
